# Randomized Double-Blind Crossover Study for Evaluating a Probiotic Mixture on Gastrointestinal and Behavioral Symptoms of Autistic Children

**DOI:** 10.3390/jcm11185263

**Published:** 2022-09-06

**Authors:** Cristina Guidetti, Elena Salvini, Maurizio Viri, Francesca Deidda, Angela Amoruso, Annalisa Visciglia, Lorenzo Drago, Matteo Calgaro, Nicola Vitulo, Marco Pane, Anna Claudia Caucino

**Affiliations:** 1 Department of Child Neuropsychiatry, University Hospital Maggiore della Carità, 28100 Novara, Italy; 2 Probiotical Research Srl, Via E. Mattei 3, 28100 Novara, Italy; 3 Department of Biomedical Sciences for Health, University of Milan, 20133 Milan, Italy; 4 Department of Biotechnology, University of Verona, 37100 Verona, Italy

**Keywords:** autism spectrum disorder, ASD, autistic children, gastrointestinal symptoms, behavioral symptoms, microbiota, microbiome analysis, gut–brain axis, probiotics, randomized double-blind crossover study

## Abstract

Autism spectrum disorders (ASDs) represent a diagnostic challenge with a still partially uncertain etiology, in which genetic and environmental factors have now been assessed. Among the hypotheses underlying the involvement of biological and environmental factors, the gut–brain axis is of particular interest in autism spectrum disorders. Several studies have highlighted the related incidence of particular gastrointestinal symptoms (GISs) in children suffering from ASDs. Probiotics have shown success in treating several gastrointestinal dysbiotic disorders; therefore, it is plausible to investigate whether they can alleviate behavioral symptoms as well. On these bases, a randomized double-blind crossover study with a placebo was conducted, evaluating the effects of a mixture of probiotics in a group of 61 subjects aged between 24 months and 16 years old with a diagnosis of ASD. Behavioral evaluation was performed through the administration of a questionnaire including a Parenting Stress Index (PSI) test and the Vineland Adaptive Behavior Scale (VABS). The Psycho-Educational Profile and the Autism Spectrum Rating Scale (ASRS) were also evaluated. Microbial composition analyses of fecal samples of the two groups was also performed. The study showed significant improvements in GISs, communication skills, maladaptive behaviors, and perceived parental stress level after the administration of probiotics. Microbiome alpha diversity was comparable between treatment arms and no significant differences were found, although beta diversity results were significantly different in the treatment group between T0 and T1 time points. *Streptococcus thermophilus*, *Bifidobacterium longum*, *Limosilactobacillus fermentum*, and *Ligilactobacillus salivarius* species were identified as some of the most discriminant taxa positively associated with T1 samples. This preliminary study corroborates the relationship between intestinal microbiota and ASD recently described in the literature.

## 1. Introduction

Autism spectrum disorder (ASD) is an early onset neurodevelopmental disorder whose etiopathogenesis is still unknown. It is characterized by impaired social communication and interaction, as well as by narrow and repetitive interests [1]. Within the diagnostic framework, different clinical conditions may coexist, making this disorder very heterogeneous. Epidemiological studies have revealed a higher incidence of ASD in males compared to females, with an average ratio of 4:1 [2,3]. In addition, etiology seems not to be attributable to cultural, socio-economic, or ethnic factors [4]. However, recent studies have evidenced a growing incidence in the last twenty years, with a total of 1% of ASD in the population [5,6]. This increase seems to be not only related to a greater effective diagnostic ability, but also to other several factors contributing to the onset of the disorder [7]. Recently, the increase in prevalence has been explained by the interaction of several factors, among which environmental influences seem to have an important role [8,9]. Furthermore, several studies have also demonstrated that subjects with ASD exhibit strong inflammatory impairment and dysregulation of the immune system [10,11,12,13,14], as well as disorders of the peripheric, enteric, and neuroimmune systems [15]. Comorbidities are also associated with other symptoms such as intellectual disability, epilepsy, feeding difficulties, and sleep disturbances [15,16,17].

Gastrointestinal symptoms (GISs) are among the most frequent comorbidities with a prevalence up to four times higher than the reference population [18]. Constipation, abdominal pain, diarrhea, flatulence, and vomiting are recurrent disorders in ASD subjects, with a variability ranging from 9% to 90% of cases [15]. Studies have highlighted the association between the presence of GISs with the severity and frequency of autistic symptoms [15,19,20].

Although a direct cause-and-effect relationship between gastrointestinal symptoms and autism spectrum disorders has not been fully demonstrated [21], extensive research showed a link between the gastrointestinal and the brain systems (gut–brain axis), and a particular bidirectional connection related to neuronal, immune, endocrine, and metabolic systems activated also by the intestinal microbiota [22,23,24]. 

The human intestine is inhabited by trillions of microorganisms, including bacteria, fungi, and viruses, which collectively are defined as intestinal microbiota, and which contribute to the health and survival of human beings [25,26]. The intestinal microbiota (or the second brain, as called by many) represents a barrier against the proliferation of opportunistic microbes, cooperates with many metabolic and neuronal processes, and neutralizes toxic substances and many ingested compounds [27]. The microbiota is also involved in the control of some signal molecules, such as norepinephrine and tryptophan, in different areas of the brain, including the cortex and the brainstem [28]. Thus, important roles as mood and behavior regulators are emerging for the intestinal microbiota. The composition of the microbiota can be influenced by various factors, including the type of birth, age, diet, environmental, and genetic influences [25,26,29]. Intestinal microbial colonization begins during the prenatal age and stabilizes at around 2–3 years of life [30,31], which is the timing of the neural development and maturation of the brain [32]. The development of the nervous system may be affected by the action of both internal and external environmental factors [33]; therefore, it is conceivable that an aberrant microbial composition (intestinal dysbiosis) can influence neurological integrity and be involved in the expression of various neuropsychiatric disorders, including ASDs [21,30,34,35]. An alteration of mucosal permeability constitutes a potential gateway for the diffusion and virulence of harmful antigens into the blood [36]. Intestinal dysbiosis is indeed involved in the reduction in the mucosal barrier and in the absorption of toxic substances. Within the human microbiota, *Firmicutes* and *Bacteroidetes* are the two most representative phyla, but at the same time they are easily affected by genetic, environmental, and neurobiological factors [37,38]. Recent studies have shown that variability in the *Firmicutes*/*Bacteroidetes* ratio can be associated with the impairment of certain cognitive and linguistic profiles, as well as with ASDs [39,40]. 

An increased intestinal permeability of the mucosal membrane was found in 43–76% of ASDs [41,42,43]. Studies comparing the intestinal flora of autistic children with gastrointestinal disorders and a control group demonstrated a change in bacterial diversity. Less diversity was correlated with a greater severity of gastrointestinal symptoms [44,45]. Additional studies revealed that patients with gastrointestinal symptoms were associated with more anxiety, aggressiveness, and stereotyped behaviors and sleep disorders, as well as a lower social interaction ability and a general state of irritability [18,22,46,47]. It has been also shown that a change in the diet, the short-term intake of antibiotics, the assumption of prebiotics and/or probiotics, or the transplantation of the fecal microbiota in subjects with ASD can rebalance the microbiota composition and improve the intestinal symptoms, including behavioral symptoms and neurological functions [48,49,50,51].

Probiotics are defined as “live microorganisms capable of bringing benefits to the health of the host” [52]. These beneficial microorganisms can reduce bacterial over-growth, stimulate the immune response (secretory IgA), regulate mucins and antioxidants, and stabilize the mucosal barrier or block and prevent the translocation of pathogens [35,53,54,55,56]. 

Some studies have highlighted the administration of probiotics in stimulating the production of oxytocin and positively influencing social behaviors [51,57,58]. A recent study demonstrated that treatment with a specific miscellanea of probiotics may decrease the severity of some ASD symptoms by improving language and communication [59]. This activity is strongly related to the type of probiotic (being strain-dependent), its concentration, the treatment duration, and the type of cohort enrolled.

Further clinical studies are necessary to understand the role of some probiotics and their long-term use on a child population with ASD.

The principal aim of the present study was to evaluate the clinical effects of a specific mixture of probiotics in children with ASD in decreasing the severity of behavioral symptoms compared with the gastrointestinal symptoms. In this regard, a randomized double-blind crossover study probiotic versus placebo was performed. Clinical evaluations were conducted for an overall follow-up of 8 months, at the time of recruitment (T0), after three months upon completion of the first probiotic or placebo cycle (T1), and at the end of the second cycle of probiotic/placebo (after the crossover) (T2). In order to observe any microbiome changes, fecal metagenomics evaluations were performed in a subgroup of patients before the probiotic treatment (T0) and after the end of first cycle of treatment (T1).

## 2. Methods and ASD Children Enrollment

Data were collected from subjects enrolled between 2016 and 2019 at the Department of Child Neuropsychiatry University Hospital Maggiore della Carità, Novara, Italy, a regional reference center, with a diagnosis of ASD.

### 2.1. Inclusion and Exclusion Criteria

Subjects aged between 24 months and 16 years old with a diagnosis of ASD (formulated between 1999 and 2019) in accordance with the DSM-IV and DSM-5 (Diagnostic and Statistical Manual of Mental Disorders: 5th Edition) were enrolled in the study. The diagnosis was performed by a multidisciplinary psychiatric and psychologist team following the national guidelines regarding the diagnostic pathway in ASDs. Diagnostic updates were periodically performed through Autism Diagnostic Observation Schedule—Second Edition (ADOS-2) and Autism Diagnostic Interview—Revised (ADI-R) tests. 

ASD children and adolescents with a previous diagnosis of organic gastrointestinal disorders (i.e., Celiac disease, Crohn’s disease, and ulcerative colitis) were excluded. Moreover, during the treatment period, all subjects who took systemic or oral long-term antibiotics (one month before and during the enrollment), or other probiotics, cortisone, anti-inflammatory drugs, amiodarone, valproate, and statins were excluded. Cases of the use of antibiotics for less than 10 days and during the enrollment were reported in the patient file.

All parents were informed that the collected data would only be used for clinical and research purposes. The study was approved by the University Hospital “Maggiore della Carità” of Novara (protocol 946/CE; study n. CE 161/15).

Due to the particular disease, all the enrolled children did not follow a balanced diet during the study; nevertheless, parents tried to limit excess consumption or any food abuse as much as possible.

**Figure 1:**
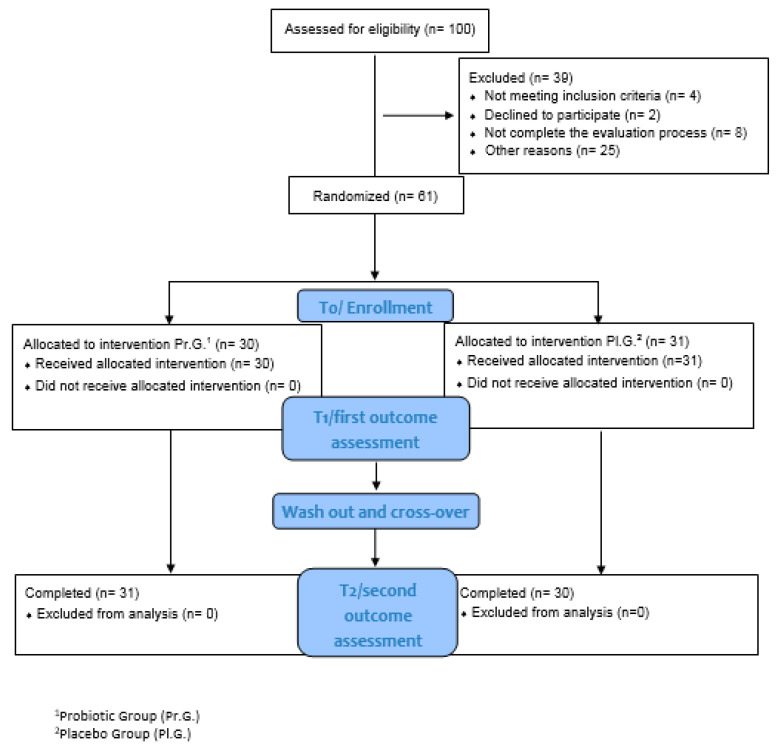
Flow chart enrollment of patients included in the study.

One hundred subjects were initially considered to be enrolled in the study. Clinical flow-chart enrollment is described in the Figure 1.

### 2.2. Study Design

A randomized double-blind crossover study probiotic versus a placebo was performed. The experimental group received one sachet of the product per day, each containing 10 × 10^9^ colony-forming unit/active fluorescent unit (CFU/AFU) 2.5 g freeze-dried powder of the probiotic mixture containing *Limosilactobacillus fermentum* LF10 (DSM 19187), *Ligilactobacillus salivarius* LS03 (DSM 22776), *Lactiplantibacillus plantarum* LP01 (LMG P-21021), and a mixture of five strains of *Bifidobacterium longum* DLBL (LF10: 4 × 10^9^ CFU/AFU/dose; LS03, LP01, and DLBL mix: 2 × 10^9^ CFU/AFU/strain/dose). The five strains of *B. longum* were: DLBL07 (DSM 25669), DLBL08 (DSM 25670), DLBL09 (DSM 25671), DLBL10 (DSM 25672), and DLBL11 (DSM 25673). In particular, *B. longum* DLBL07, DLB10, and DLBL11 strains have demonstrated an antiflammatory in vitro activity and a high homeostatic intestinal activity (data not shown).

The mixture comprised commercially available probiotic strains extensively used for food supplement formulations in Probiotical S.p.A., Novara, Italy.

The choice of strains was based on their functional activities; specifically, *L. fermentum* LF10 exerted a contrasting action against yeasts mainly belonging to the *Candida* genus; *L. salivarius* LS03 exerted a strong oxidative stress reduction activity; *L. plantarum* LP01 demonstrated a relevant anti-inflammatory activity and an inhibitory activity against coliforms, with particular reference to *E. coli*; the *B. longum* DLBL mix, isolated from healthy donors over one hundred years old, exerted in vitro activity on cytokine regulation (data not shown). The placebo group received sachets containing 2.5 g of maltodextrin in powder form. The placebo powder was indistinguishable from the probiotics powder in color, taste, and smell, but contained no probiotic bacteria. Participants were instructed to dissolve the powder in water or milk and drink it in the morning with breakfast.

**Figure 2:**
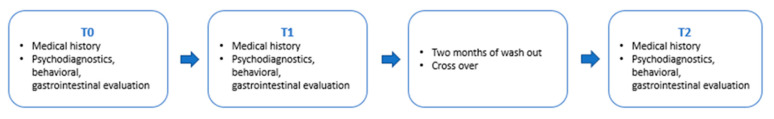
Description of treatments and follow-up.

The study had an overall follow-up of 8 months. Clinical evaluations at the time of recruitment (T0), after three months upon completion of the first probiotic or placebo cycle (T1), and at the end of the second cycle of probiotic/placebo (after the crossover) (T2) were performed. Two months of washout elapsed between the first and second treatment cycles. Fecal samples were collected from 24 patients at time points T0 and T1 (Figure 2). 

### 2.3. Data Collection, Clinical Evaluations, and Probiotic/Placebo Administration at the Time Points

At time T0, medical and gastrointestinal history, as well as evaluations performed through the GI Severity Index [26] (a sum of each clinical variable, scored from 0 to 2) were assessed. Behavioral evaluations were performed through the administration of a questionnaire, including a Parenting Stress Index (PSI) test (the sum of 36 clinical variables included, each scored from 1 to 5 on a Vineland Adaptive Behavior Scale (VABS) (compound score M = 100; s.d. = 15) [60,61]. The Psycho-Educational Profile (PEP 3rd edition), which includes 172 items with 3 different scale-scores, and the Autism Spectrum Rating Scales (ASRS) were also included [62,63].

The first cycle of probiotics/placebo was then delivered to the families with the following regimen: two sachets a day 30 min before meals or 3 h after meals to be administered for one month followed by one sachet a day for two months.

At the end of the first cycle of treatment (T1), psychodiagnostics, behavioral, and gastrointestinal evaluations were performed. Two months of washout of probiotic/placebo started after this visit. At T2, the same clinical evaluation was then conducted.

### 2.4. Statistical Method

Continuous data are reported as the median (I, III quartiles); categorical data are reported as a percentage and absolute frequencies. Wilcoxon-type tests were performed for continuous variables, and the Pearson chi-squared test or Fisher-exact test, whichever was appropriate, were applied for categorical variables.

The study outcomes were analyzed via the generalized linear mixed model (GLMM) method. The estimated model included, as covariates:

- The treatment effects;
- The time of treatment administration effect among treatment sequences (T0, T1, and T2);
- The carry-over (sequence) effect computed including an interaction term between time and treatment in the model;
- A random effect term (random intercept) on the patient’s identification code, accounting for correlations among repeated measures of the same patient.

The GLMMs were also computed, considering outcome variations until the end of the follow-up period as the endpoint (T2–T0, T2–T1).

A two-tailed test was considered for the hypothesis testing procedure, and statistically significant values were considered to reach a *p*-value < 0.05. Statistical analyses were conducted using R 3.5.2 [64] with rms [65] packages.

### 2.5. Fecal Sample DNA Extraction and Metagenome Sequencing

DNA isolation was performed using the Pure Link Microbiome DNA Purification Kit (Thermo Fisher Scientific, Monza, Italy) according to the manufacturer’s protocol. A stool sample was scooped on dry ice, transferred to an Eppendorf tube, and weighed. Approximately 200 mg of stool sample was used. DNA was eluted then measured with Qbit using Qubit ds DNS HS Assay Kit Molecular Probes (Thermo Fisher Scientific) and stored in elution buffer at −20 °C until use in PCR reactions. 

The 16S region was amplified with a 16S Ion Metagenomics Kit™ (Life Technologies, Grand Island, NY, USA) by two separate PCR reactions using primer sets V2, V4, V8 and V3, V6-7, and V9. Equal volumes of V2, V4, V8 and V3, V6-7, and V9 amplification reactions were combined. Fifty nanograms of combined amplicons was processed to develop the DNA library using an Ion Plus Fragment Library Kit™ and Ion Xpress Barcodes Adapters, 1–32™ (Life Technologies, Grand Island, NY, USA). Adapter-ligated and nick-repaired DNA was amplified through the following steps: 1 cycle of 95 °C for 5 min; 5 cycles of 95 °C for 15 s, 58 °C for 15 s, 70 °C for 1 min; hold at 4 °C. Each step was followed by purification using 1.4 volumes of Agencourt AM Pure beads (Beckman Coulter, Inc, Atlanta, Georgia) and eluted in a low-Tris–EDTA buffer. The concentration of each 16S library was determined with a Qubit fluorometer. The library was diluted to ~100 pM prior to template preparation. Equal volumes of samples (for a maximum of 24 samples) were combined and processed with a Hi-Q Chef Kit, using the Chef System, according to the manufacturer’ instructions. Sequencing was performed on the Ion Personal Genome Machine (PGM) using a 400 bp kit and a 318 v2 chip.

Base calling and run demultiplexing were performed in Torrent Suite (Life Technologies, Grand Island, NY, USA) with default parameters. The mean read length for both forward and reverse reads ranged between 235 bp and 238 bp for all four samples. 

### 2.6. Bioinformatics Data Analysis

Sequencing reads were processed using the workflow implemented in the IonReporter cloud server (Metagenomics 16S w1.1 version located in Novara, Italy). In the first step of the workflow, the reads were trimmed by primer and length. When trimming, the primer search used the 15 bases closest to the sequence itself, allowing a maximum of three mismatches. After primer trimming, each sequence was trimmed to the closest 0 bp point to speed up the clustering later in the process. For example, if a sequence was 187 bases long, it was trimmed to 180 bp. After primer trimming and length check, identical reads were considered just once, together with their abundance/copy number. Quick filtering removed all unique reads with a copy number lower than the user-set threshold. By default, the threshold was set to 10 reads. The metagenomics workflow provides access to two reference databases for mapping: the curated MicroSEQTM ID database and the curated GreenGenes database. During the analysis, both databases were selected. With this option, the reads were first mapped against the curated MicroSEQTM db. Reads that were not mapped against this database were mapped against the curated GreenGenes database. The mapping was performed using a BLAST algorithm, setting an e-value cutoff lower than 0.01.

A consensus taxa count matrix table, derived from the cumulative sum of the count of each taxon identified by the different hypervariable regions, was produced by the workflow. For the downstream analysis, the count matrix with taxa annotated at species level was considered.

The phyloseq package was used to perform all the downstream analyses in the R environment [66].

### 2.7. Data Quality Assessment and Filtering

Rarefaction curves on raw data were evaluated to assess the species richness among samples as a function of the sequencing depth. Data were pre-processed by filtering taxa with a low average relative abundance, setting a threshold of 0.005%. Furthermore, taxa present in fewer than two samples were discarded. Phylum members of *Synergistetes* (cumulative relative abundance = 0.000004%) were discarded by this filter. The pre-processing output counts were then transformed to relative abundances, and the 10 most present genera were plotted to phylum level.

### 2.8. Biodiversity Measurements

The Shannon–Wiener index was used to calculate α-diversity, which was plotted stratifying the samples according to treatment arm (probiotic vs. placebo) and time points (T0 and T1). Mann–Whitney tests were performed to verify statistical differences in the α-diversity across T0 and T1 time points. To measure β-diversity, data were normalized using total sum scaling (TSS) normalization through the *phyloseq_standardize_otu_abundance* function of the vmikk/metagMisc package (github.com/vmikk/metagMisc, accessed on 20 June 2022). Bray–Curtis distance metrics were used to measure diversity between sample counts, and the principal coordinate analysis (PCoA) ordination method was used to ordinate the samples in a reduced dimensional space using the ordinate function of the Vegan package (v2.6-2) [67]. To test the multivariate homogeneity of group dispersions, the *betadisper* function was used. Finally, the permutational analysis of variance (PERMANOVA) was performed, using the *adonis2* function, to investigate treatment arms and time point contributions on the beta diversity variability. 

### 2.9. Biomarker’s Identification

A discriminant analysis was computed using sPLS-DA (sparse partial least square discriminant Analysis) methods to identify possible biomarkers associated with time points T0 and T1 in the two treatment arms (probiotic-treated and placebo).

In particular, following the default mixOmics (v6.20) [68,69] pipeline (http://mixomics.org/case-studies/splsda-srbct/, accessed on 23 June 2022), a pseudo-count value of 1 was added to the raw counts, which were then normalized with TSS and centered log-ratio (CLR)-transformed. This compositional approach is based on the centered log-ratio (CLR) value, which is computed through the ratio of a taxon’s abundance, and the geometric mean of all the other taxa abundances in the sample. A positive (or negative) value of the CLR indicates that the abundance of the considered taxon is CLR-fold bigger (or smaller) than the geometric mean of the abundances of all the taxa. Consequently, a zero value does not indicate the absence; instead, it indicates that the difference between the taxa abundance and the geometric mean of the abundances is null. 

The sPLS-DA classification performance was measured with respect to the number of selected variables in the model with the function *tune. splsda*. The tuning was performed with a leave-one-out cross validation (CV) process, and a prediction distance (maximal distance) was chosen to predict class membership across all CV runs. The ability of the model to correctly classify samples was summarized by the classification accuracy, which was the mean proportion of samples that were correctly classified in the CV process.

For each comparison, a summary image was plotted using the HotLoadings function (github.com/mcalgaro93/HotLoadings accessed on 23 June 2022), displaying the discriminant taxa loadings and the related heatmap.

## 3. Results

### 3.1. Microbial Composition of the Samples

**Figure 3:**
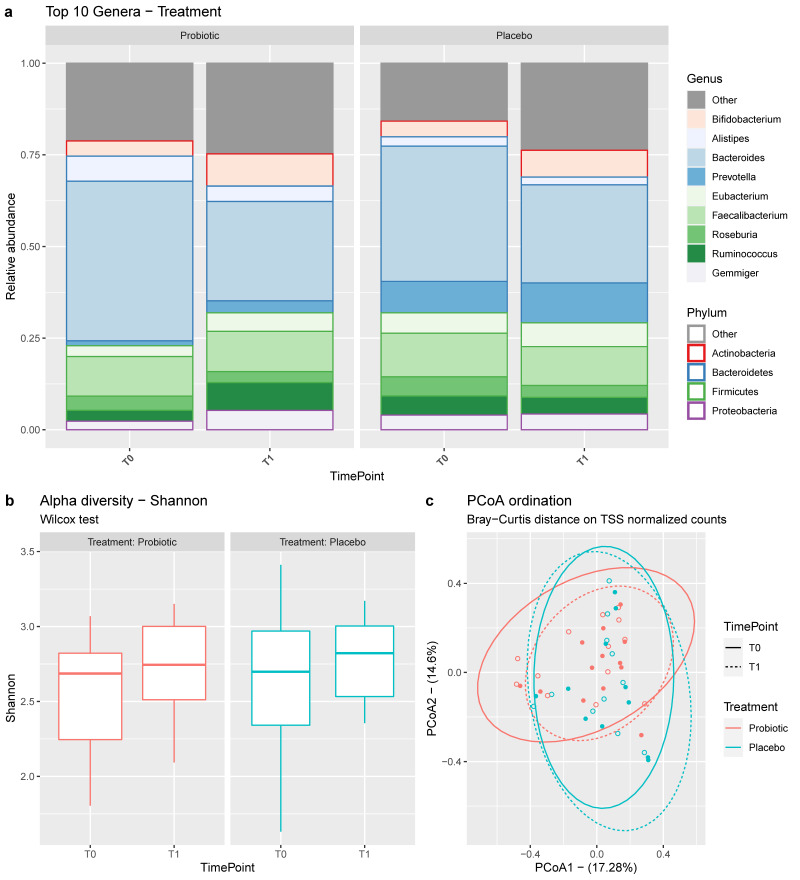
(**a**) The top 10 genera and related phyla of membership. Genera are plotted for their mean relative abundance over time point, faceted by treatment arm. (**b**) Shannon–Wiener α-diversity, over time point and faceted by treatment arm. (**c**) β-diversity bidimensional representation (PCoA ordination method on the Bray–Curtis distance matrix of TSS normalized counts). Colored by treatment arm and shaped by time point.

Stool samples were collected for 24 subjects (13 probiotic treated, 11 placebo) at time points T0 and T1 for a total of 48 samples. The 16S rRNA metabarcoding analysis resulted in 157 taxa with a median of 83901 bacterial reads (IQR 60646–114393) per sample retained after data processing, quality control, and filtering (Figures S1–S4). *Bacteroidetes*, *Firmicutes*, and *Actinobacteria* were the most abundant phyla, with more than 90% of the total counts, followed by *Proteobacteria* and *Verrucomicrobia*. *Bacteroides* and *Faecalibacterium* were the two most abundant genera, contributing to 45% of the total counts, followed by *Bifidobacterium*, *Roseburia*, *Gemmiger*, *Alistipes*, and other genera (Figure 3).

Alpha diversity was comparable between treatment and placebo arms, and no significant differences were found between time points (Figure 3b).

Starting from the beta diversity (Figure 3c), a permutational analysis of variance (PERMANOVA) was performed. This analysis is a permutational and multivariate approach that enables testing of both the homogeneity of group dispersions (variance) and the distance between group centroids. As desirable, the null hypothesis that both treatment and placebo groups had the same dispersions between T0 and T1 time points (*p* > 0.1) was accepted. Interestingly, the treatment group exhibited significantly different results between T0 and T1 time points (*p* = 0.019). This difference was not observed in the placebo group (*p* = 0.628).

### 3.2. Biomarker’s Identification

Multivariate analysis based on sPLS-DA was performed to better characterize the differences in the microbial compositions between T0 and T1 samples for each treatment arm. This compositional approach is based on the CLR values that indicate the abundance of one single taxon relative to the average (geometric mean) abundance of all the other taxa in the sample (further details can be found in the Methods section).

**Figure 4:**
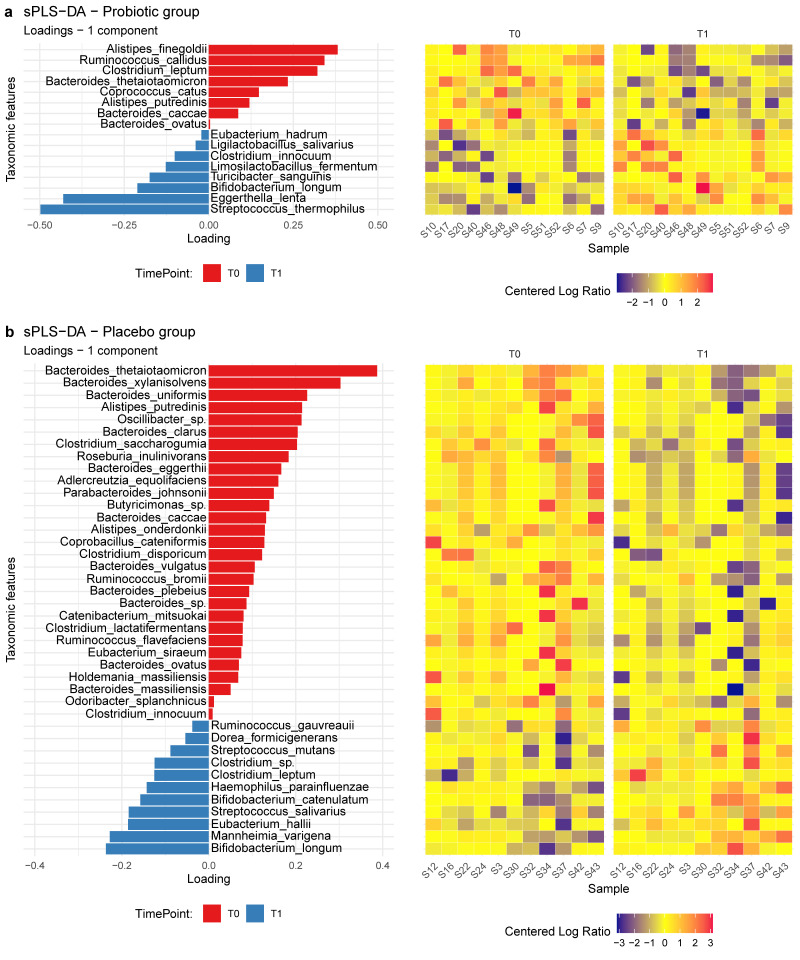
(**a**,**b**)**.** sPLS-DA. Loading values (on the left) represent the discriminant taxa associated with the samples at time point T0. The bigger the loading absolute value, the stronger the association. Heatmap (on the right) shows the CLR values of the discriminant taxa in all the samples. (**a**) Probiotic treatment group. (**b**) Placebo treatment group.

The analysis revealed that a group of 16 ASVs was able to discriminate between T0 and T1 samples for the treatment group with a classification accuracy of 93%. Eight of them were positively associated with time point T0, whereas the remaining eight were positively associated with time point T1. Among these, *Streptococcus thermophilus*, *Bifidobacterium longum*, *Limosilactobacillus fermentum*, and *Ligilactobacillus salivarius* species were found to be some of the most discriminant taxa positively associated with T1 samples (Figure 4a).

On the other side, a group of 40 ASVs was needed to discriminate between T0 and T1 samples for the placebo group with a lower classification accuracy of 64%: 29 out of 40 were associated with the time point T0, whereas the remaining 11 were associated with time point T1. The most discriminant ASV, positively associated with time point T1, belonged to the *Bifidobacterium longum* species (Figure 4b).

### 3.3. Clinical Data

The patient’s baseline characteristics are summarized in Table 1. 

From an initial sample of 100 patients the following were excluded: 2 children who had a comorbid diagnosis of gastrointestinal disorders of an organic nature and were already following probiotic therapy; 2 because they were taking drugs incompatible with the study; then, there were 2 further dropouts after 10 days from the start of treatment.

A total of 94 patients met the study’s inclusion criteria. Of these, 25 subjects abandoned the study at various times, because they became unavailable, took other probiotics during the experimentation, were unable to take the product due to the palatability of the mixture, or asked to leave the study for unspecified reasons. Finally, eight subjects did not complete the evaluation process.

Three children among those recruited exhibited a worsening in gastrointestinal symptoms in the first week of administration of the probiotic, and then stabilized.

Sixty-one subjects completed the study.

Follow-ups were completed by 61 patients, 30 children who were randomized in the probiotic-treated group and 31 in the placebo group. 

Of the sample, 82% was female (50 participants in total). The gender composition was balanced across the randomization groups. The median age of the sample was 48 months and was not significantly different across the groups: 49.5 months for the probiotic group, and 47 for the placebo group (Table 1). 

The randomization procedure ensured the balance of patient characteristics across the samples. Table 1 presents no differences in the baseline features considering all the baseline characteristics, including the presenting therapy, symptoms of allergy, and the genetic characteristics together with the demographic child profile (gender and age). 

The study outcome assessments are presented in Table 2, reporting the outcome-specific treatment effects with the standard error (SE) and *p*-value. A significant treatment effect was shown for the child’s specific physical and cognitive function. 

The GLMM estimate indicated an improvement in the children’s diarrhea symptoms, together with improvements in receptive language scale (Table 2, Panel A).

The administered therapy involved a significant improvement in parent’s distress symptoms. In particular, this was evidenced in the paternal and maternal PSSI scores improving, as well as the paternal overall distress score (Table 2, Panel B).

The outcome assessment was also performed considering the variations until the end of the follow-up period (T2–T0, T2–T1). Table 2 (Panel B) evidences a significant reduction in gastrointestinal symptoms such as abdominal pain and gain in behavioral outcomes on the Vineland-I communication attitude and Disadaptive Behaviors (PEP3).

## 4. Discussion

Gastrointestinal abnormalities in ASD seem related to the dysbiosis and the “inflammation hypothesis”. A wide range of GISs have been reported in ASD children, such as diarrhea, constipation, vomiting, feeding problems, reflux, and abdominal pain. Whether the changes in the gut microbiota and dysbiotic status are caused by gastrointestinal disturbances or whether these microbiota impairments cause the gastrointestinal symptoms in ASDs are still under investigation. 

Due to the emerging role of gut dysbiosis in ASD, studies have focused on rebalancing the gut microbiota as a possible therapeutic approach, using dietary and/or oral probiotics. This randomized double-blind crossover placebo study, which involved 61 children with ASD, showed that the administration of specific probiotics can partially but significantly reduce the severity of behavioral symptoms related to the gastrointestinal ones. Namely, these statistically significant results included a general improvement in gastrointestinal symptoms, as well as the socio-relational aspect, the maladaptive behaviors, and the perceived parental stress level. The presence of taxa related to *Streptococcus thermophilus*, *Bifidobacterium longum*, *Limosilactobacillus fermentum*, and *Ligilactobacillus salivarius* species in the samples of the probiotic group at time point T1 is coherent with the probiotic formulation. Similarly, the presence of taxa related to *Bifidobacterium longum* species (and the absence of the abovementioned species), associated with the samples after the placebo supplementation is coherent with the known bifidogenic effect of maltodextrins, which comprise the placebo arm. These results agree with the findings of other authors, such as Dinan et al. [57], Higashida et al. [70], Meyer-Lindenberg et al. [71], and Shahrestani et al. [72], who demonstrated the positive influence of probiotics on social behaviors. Other studies [59,73,74,75] reported an improvement in maladaptive behaviors in children with ASD after the intake of probiotics. A study by Santocchi E. et al. [76] evidenced statistically significant improvements in adaptive functions in children diagnosed with ASD with gastrointestinal symptoms. The authors hypothesized that this improvement may be associated with both the improvement in gastrointestinal symptoms and multisensory processing associated with the serotonergic system modulated by improvements in the intestinal microbiota [77]. In contrast, other studies [78,79] have not revealed significant changes in the maladaptive behaviors after oral probiotics. These discrepancies can be due to the heterogeneity of the studies or to the use of different probiotic strains, this activity being strain-dependent.

Our results have demonstrated a statistically significant improvement in the probiotic group of abdominal pain and diarrhea episodes, which is in line with other similar studies [33,59,75]. Other studies also revealed [59,73,75,76,78] that gastrointestinal symptoms can be improved after the consumption of a prebiotic and probiotic, or a gluten- and casein-free diet [51,78], demonstrating that dietary interventions can have positive effects on the gut–brain axis in ASDs.

Our study indeed showed significant improvements in communication skills and receptive language in the probiotic-treated group, which is in accordance with a previous report [76].

Similar positive outcomes have been obtained in the parental stress test, or in the Difficult Child subscale evaluation (focused on temperamental characteristics and challenging, disobedient, and demanding behaviors). 

No statistically significant differences between probiotic and placebo groups have been observed regarding sensory sensitivity evaluated by means of an ASRS questionnaire.

Taken together, our results confirm the general improvements in gastrointestinal and behavioral symptoms in children with ASD, as well as the decrease in perceived parental stress, after probiotic oral administration.

The double-blind crossover tailored study, the rigorousness of clinical evaluations, and the control of the carry-over effect according to the GLMM procedures indicate the strength of the study. 

Among the limits, the need to have water-soluble probiotic/placebo for those children in whom food selectivity is often an issue surely influenced the number of dropouts.

A further limitation may include the lack of subgrouping ASD patients in presenting GI symptoms or not. Bowel dysfunctions are generally harbored by the majority of children diagnosed with ASD, but not in all cases of autism. 

The use of self-report tools (ASRS and PSSI) and parent-report tools (GSI and Vineland) can have intrinsic limits as well, due to the lack of adequate inter-rater and test-retest reliability. In addition, these evaluations may be influenced by the possible placebo effect in parental perception [80].

These limitations are in any case overtaken by a large number of patients enrolled which, although they may seem few in number, it is not easy to enroll, harmonize, and homogenize 100 patients in order to have 61 evaluable ASDs.

A larger ASD population at national or international level would be needed for better understanding these fine mechanisms involving the gut–brain axis.

## 5. Conclusions

In conclusion, this randomized double-blind cross-over placebo study, which actively involved 61 children with ASD, showed that the administration of specific probiotics can partially but significantly reduce the severity of behavioral (socio-relational aspect, the maladaptive behaviors, and the perceived parental stress level) as well as the gastrointestinal symptoms which generally affects these type of patients. The presence of those taxa related to *Streptococcus thermophilus*, *Bifidobacterium longum*, *Limosilactobacillus fermentum*, and *Ligilactobacillus salivarius* species in the fecal samples may represent a huge probiotics intestinal colonization in these AD patients.

The obtained results emphasize the importance of continuing research on the potentially positive effects of probiotics on the symptoms of autism spectrum disorders, in order to better understand the gut–brain connection in ASD.

## Figures and Tables

**Table 1:** Descriptive statistics. Continuous data are reported as the median (I, III quartiles); categorical data are reported as a percentage and absolute frequencies. Wilcoxon-type tests were performed for continuous variables, and the Pearson chi-squared test or Fisher’s exact test were conducted for categorical variables, wherever appropriate.

Variable	N	Probiotic Group	Placebo Group	Overall	*p* Value
	-	(N = 30)	(N = 31)	(N = 61)	
Age	61	40.2/49.5/71.5	37.5/47.0/58.0	39.0/48.0/65.0	0.44
Gender: 0	61	20% (6)	16% (5)	18% (11)	0.694
1	-	80% (24)	84% (26)	82% (50)	
Childbirth: 0	61	70% (21)	74% (23)	72% (44)	0.715
1	-	30% (9)	26% (8)	28% (17)	
Breastfeeding: 0	60	40% (12)	70% (21)	55% (33)	0.063
1	-	23% (7)	10% (3)	17% (10)	
2	-	37% (11)	20% (6)	28% (17)	
Intolerances: 0	61	90% (27)	87% (27)	89% (54)	0.722
1	-	10% (3)	13% (4)	11% (7)	
Rehabilitation. therapy: 0	61	10% (3)	6% (2)	8% (5)	0.614
1	-	90% (27)	94% (29)	92% (56)	
Ages. Diagnosis	60	30.2/37.0/50.5 41.0+/−15.9	30.2/37.5/48.8 40.4+/−12.5	30.0/37.5/49.2 40.7+/−14.2	0.942
Karyotype: 1	7	100% (5)	100% (2)	100% (7)	
0	-	0% (0)	0% (0)	0% (0)	
X. fragile: 0	39	100% (17)	95% (21)	97% (38)	0.373
1	-	0% (0)	5% (1)	3% (1)	
CGH. array: 0	45	90% (19)	79% (19)	84% (38)	0.296
1	-	10% (2)	21% (5)	16% (7)	
Epilessia: 0	55	96% (25)	100% (29)	98% (54)	0.286
1	-	4% (1)	0% (0)	2% (1)	
MRI: 0	45	12% (3)	24% (5)	18% (8)	0.322
1	-	88% (21)	76% (16)	82% (37)	
AA.plasmatic.acids.organic.urinary: 0	48	5% (1)	12% (3)	8% (4)	0.382
1	-	95% (21)	88% (23)	92% (44)	
Verbal: 0	61	53% (16)	52% (16)	52% (32)	0.893
1	-	47% (14)	48% (15)	48% (29)	
Other diagnosis in. brothers sisters: 0	60	80% (24)	83% (25)	82% (49)	0.739
1	-	20% (6)	17% (5)	18% (11)	
Pharmacological therapy: 0	61	90% (27)	97% (30)	93% (57)	0.285
1	-	10% (3)	3% (1)	7% (4)	

**Table 2:** Generalized linear mixed model treatment effect estimate (GLMM) with standard error (SE) and p-values. Panel A reports the child’s specific outcomes; panel B shows the parental outcomes; and Panel C details the delta outcomes.

	Panel A Child Absolute Value		
	Estimate (Treatment vs. Placebo)	Standard Error	*p* Value
**Diarrhea**	−0.15	0.07	0.05
**Receptive language**	18	8.44	0.03
	**Panel B Parent absolute value**		
	**Estimate (Treatment vs. placebo)**	**Standard Error**	***p*** **value**
**PSSI.mother**	−18.9	8.75	0.03
**PSSI.father.DC**	−15.42	7.21	0.04
**PSSI.father.total Stress**	−18.9	7.68	0.01
	**Panel C Child variation**		
	**Estimate (Treatment vs. placebo)**	**Standard Error**	***p*** **value**
**Abdominal pain**	−0.28	0.15	0.05
**Communication (Vineland)**	11.3	4.6	0.01
**Disadaptive behaviors (PEP3)**	8.59	4.31	0.05

## Supplementary Materials

The following supporting information can be downloaded at: https://www.mdpi.com/article/10.3390/jcm11185263/s1, Figure S1: Sequencing depth; Figure S2: Rarefaction curves; Figure S3: Pre-filtering prevalence; Figure S4: Post-filtering prevalence.

## References

[1] American Psychiatric Association (2013). DSM-5 Task Force. Diagnostic and Statistical Manual of Mental Disorders: DSM-5.

[2] Loomers R., Hull L., Mandy W.P.L. (2017). What is the male-to-female ratio in autism spectrum disorder? A systematic review and meta-analysis. J. Am. Acad. Child Adolesc. Psychiatry.

[3] Brugha T.S., Spiers N., Bankart J., Cooper S.-A., McManus S., Gullon-Scott F., Smith J., Tyrer F. (2016). epidemiology of autism in adults across age groups and ability levels. Br. J. Psychiatry.

[4] Elsabbagh M., Divan G., Koh Y.-J., Kim Y.S., Kauchali S., Marcín C., Montiel-Nava C., Patel V., Paula C.S., Wang C. (2012). Global prevalence of autism and other pervasive developmental disorders. Autism Res..

[5] Baxter A.J., Brugha T., Erskine H., Scheurer R., Vos T., Scott J. (2015). The epidemiology and global burden of autism spectrum disorders. Psychol. Med..

[6] Centers for Disease Control and Prevention Data & Statistics on Autism Spectrum Disorder. January 2019. https://www.cdc.gov/ncbddd/autism/data.html.

[7] Tordjman S., Somogyi E., Coulon N., Kermarrec S., Cohen D., Bronsard G., Bonnot O., Weismann-Arcache C., Botbol M., Lauth B. (2014). Gene × Environment interactions in autism spectrum disorders: Role of epigenetic mechanisms. Front. Psychiatry.

[8] Bölte S.B., Girdler S., Marschik P.B. (2019). The contribution of environmental exposure to the etiology of autism spectrum disorder. Cell. Mol. Life Sci..

[9] Alibek K., Farmer S., Tskhay A., Moldakozhayev A., Alibek K., Isakov T. (2019). The Role of Infection, Inflammation and Genetic Alterations in ASD Etiopathogenesis: A Review. J. Neurol. Psychiatr. Disord..

[10] Fakhoury M. (2015). Autistic spectrum disorders: A review of clinical features, theories and diagnosis. Int. J. Dev. Neurosci..

[11] Jyonouchi H., Sun S., Itokazu N. (2002). Innate immunity associated with inflammatory responses and cytokine production against common dietary proteins in patients with autism spectrum disorder. Neuropsychobiology.

[12] Neale B.M., Kou Y., Liu L., Ma’ayan A., Samocha K.E., Sabo A., Lin C.-F., Stevens C., Wang L.-S., Makarov V. (2012). Patterns and rates of exonic de novo mutations in autism spectrum disorders. Nature.

[13] Newschaffer C.J., Croen L.A., Daniels J., Giarelli E., Grether J.K., Levy S.E., Mandell D.S., Miller L.A., Pinto-Martin J., Reaven J. (2007). The epidemiology of autism spectrum disorders. Annu. Rev. Public Health.

[14] Rossignol D.A., Frye R.E. (2012). A review of research trends in physiological abnormalities in autism spectrum disorders: Immune dysregulation, inflammation, oxidative stress, mitochondrial dysfunction and enviromental toxicant exposures. Mol. Psychiatry.

[15] Voung H.E., Hsiao E.Y. (2017). Emerging roles for the gut microbiome in autism spectrum disorder. Biol Psychiatry..

[16] Marshall J., Hill R.J., Ziviani J., Dodrill P. (2014). Features of feeding difficulty in children with Autism Spectrum Disorder. Int. J. Speech. Lang. Pathol..

[17] Devnani P.A., Hegde A.U. (2015). Autism and sleep disorders. J. Pediatr. Neurosci..

[18] Marler S., Ferguson B.J., Lee E.B., Peters B., Williams K.C., McDonnell E., Macklin E.A., Levitt P., Margolis K.G., Beversdorf D.Q. (2017). Association of Rigid-Compulsive Behavior with Functional Constipation in Autism Spectrum Disorder. J. Autism Dev. Disord..

[19] Adams J.B., Johansen L.J., Powell L.D., Quig D., Rubin R.A. (2011). Gastrointestinal flora and gastrointestinal status in children with autism-comparisons to typical children and correlation with autism severity. BMC Gastroenterol..

[20] Kang D.W., Adams J.B., Gregory A.C., Borody T., Chittick L., Fasano A., Khoruts A., Geis E., Maldonado J., McDonough-Means S. (2017). Microbiota Transfer Therapy alters gut ecosystem and improves gastrointestinal and autism symptoms: An open-label study. Microbiome.

[21] Mayer E.A., Padua D., Tillisch K. (2014). Altered brain-gut axis in autism: Comorbidity or causative machanisms?. Bioessays.

[22] Iovene M.R., Bombace F., Maresca R., Sapone A., Iardino P., Picardi A., Marotta R., Schiraldi C., Siniscalco D., Serra N. (2017). Intestinal Dysbiosis and Yeast isolation in stool of subjects with autism spectrum disorders. Mycopathologia.

[23] Martin C.R., Osadchiy V., Kalani A., Mayer E.A. (2018). The Brain-Gut-Microbiome Axis. Cell. Mol. Gastroenterol. Hepatol..

[24] Sekirov I., Russell S.L., Caetano L., Antunes M., Brett Finlay B. (2010). Gut microbiota in health and disease. Physiol. Rev..

[25] Clemente J.C., Ursell L.K., Parfrey L.W., Knight R. (2012). The impact of the gut microbiota on human health: An integrative view. Cell.

[26] Schneider C.K., Melmed R.D., Barstow L.E., Enriquez F.J., Ranger-Moore J., Ostrem J.A. (2006). Oral human immunoglobulin for children with autism and gastrointestinal dysfunction: A prospective, open-label study. J. Autism Dev. Disord..

[27] Umbrello G., Esposito S. (2016). Microbiota and neurologic diseases: Potential effects of probiotics. J. Transl. Med..

[28] Forsythe P., Sudo N., Dinan T., Taylor V.H., Bienenstock J. (2009). Mood and gut feelings. Brain Behav. Immun..

[29] Thursby E., Juge N. (2017). Introduction to the human gut microbiota. Biochem. J..

[30] Buie T. (2015). Potential etiologic factors of microbiome disruption in autism. Clin. Ther..

[31] Borre Y.E., Moloney R.D., Clarke G., Dinan T.G., Cyran J.F. (2014). The impact of microbiota on brain and behavior: Machanisms &therapeutic potential. Adv. Exp. Med. Biol..

[32] Diaz Heijtz R. (2016). Fetal, neonatal, and infant microbiome: Perturbations and subsequent effects on brain development and behavior. Semin. Fetal. Neonatal. Med..

[33] Srikantha P., Mohajeri M.H. (2019). The possible role of the microbiota-gut-brain-axis in autism spectrum disorder. Int. J. Mol. Sci..

[34] Cryan J.F., Dinan T.G. (2012). Mind-altering microorganisms: The impact of the gut microbiota on brain and behaviour. Nat. Rev. Neurosci..

[35] Fond G., Boukouaci W., Chevalier G., Regnault A., Eberl G., Hamdani N., Dickerson F., Macgregor A., Boyer L., Dargel A. (2015). The “psychomicrobiotic”: Targeting microbiota in major psychiatric disorders: A systematic review. Pathol. Biol..

[36] Alonso C., Vicario M., Pigrau M., Lobo B., Santos J. (2014). Intestinal barrier function and the brain-gut axis. Adv. Exp. Med. Biol..

[37] Ho L.K.H., Tong V.J.W., Syn N., Nagarajan N., Tham E.H., Tay S.K., Shofey S., Tambyah P.A., Law E.C.N. (2020). Gut microbiota changes in children with autism spectrum disorder: A systematic review. Gut Pathog..

[38] Gill S., Pop M., Deboy R., Eckburg P., Turnbaugh P., Samuel B., Gordon J.I., Relman D.A., Fraser-Liggett C.M., Nelson K.E. (2006). Metagenomic analysis of the human distal gut microbiome. Science.

[39] Carlson A.L., Xia K., Azcate-Peril M.A., Goldman B.D., Ahn M., Styner M.A., Thompson A.L., Geng X., Gilmore J.H., Knickmeyer R.C. (2018). Infant gut microbiome associated with cognitive development. Biol. Psychiatry.

[40] Borre Y., O’Keeffe G., Clarke G., Stanton C., Dinan T., Cryan J. (2014). Microbiota and neurodevelopmental windows: Implications for brain disorders. Trends Mol. Med..

[41] Emanuele E., Orsi P., Boso M., Broglia D., Brondino N., Barale F., di Nemi S.U., Politi P. (2010). Low-grade endotoxemia in patients with severe autism. Neurosci. Lett..

[42] Kunze W.A., Bornstein J.C., Furness J.B. (1995). Identification of sensory nerve cells in a peripheral organ (the intestine) of mammal. Neuroscience.

[43] Perez-Burgos A., Mao Y.K., Bienenstock J., Kunze W.A. (2014). The gut-brain axis rewired: Adding a functional vagal nicotinic “sensory synapse”. FASEB J..

[44] Kang D.W., Park J.G., Ilhan Z.E., Wallstrom G., Labaer J.B., Krajmalnik-Brown R. (2013). Reduced incidence of Prevotella and other fermenters in intestinal microflora of autistic children. PLoS ONE.

[45] Finegold S.M., Summanen P.H., Downes J., Corbett K., Komoriya T. (2017). Detection of Clostridum perfringens toxin genes in the gut microbiota of autistic children. Anaerobe.

[46] Fulceri F., Morelli M., Santocchi E., Cena H., Del Bianco T., Narzisi S., Calderoni S., Muratori F. (2016). Gastrointestinal Symptoms and behavioral problems in preschoolers with autism spectrum disorder. Dig. Liver Dis..

[47] Buie T., Campbell D.B., Fuchs G.J., Furuta G.T., Levy J., Vandewater J., Whitaker A.H., Atkins D., Bauman M.L., Beaudet A.L. (2010). Evaluation, diagnosis, and treatment of gastrointestinal disorders in individuals with ASDs: A consensus report. Pediatrics.

[48] Xu M., Xu X., Li J., Li F. (2019). Association between gut microbiota and autism spectrum disorder: A systematic review and meta-analysis. Front. Psychiatry.

[49] Luna R.A., Savidge T.C., Williams K.C. (2016). The Brain-Gut-Microbiome Axis: What Role Does it Play in Autism Spectrum Disorder?. Curr. Dev. Disord. Rep..

[50] Fattorusso A., Di Genova L., Dell’isola G.B., Mencaroni E., Esposito S. (2019). Autism spectrum disorders and the gut microbiota. Nutrients.

[51] Grimaldi R., Gibson G.R., Vulevic J., Giallourou N., Castro-Mejía J.L., Hansen L.H., Leigh Gibson E., Nielsen D.S., Costabile A. (2018). A prebiotic intervention study in children with autism spectrum disorders (ASDs). Microbiome.

[52] Navarro F., Liu Y., Rhoads J.M. (2016). Can probiotics benefit children autism spectrum disorders?. World J Gastroenterol..

[53] Van Minnen L.P., Timmerman H.M., Lutgendorff F., Verheem A., Harmsen W., Konstantinov S.R., Smidt H., Visser M.R., Rijkers G.T., Gooszen H.G. (2007). SurModification of intestinal flora with multispecies probiotics reduces bacterial translocation and improves clinical course in a rat model of acute pancreatitis. Surgery.

[54] Lutgendorff F., Akkermans L.M., Soderholm J.D. (2008). The role of microbiota and probiotics in stress-induced gastrointestinal damage. Curr. Mol. Med..

[55] Lutgendorff F., Nijmeijer R.M., Sandstrom P.A., Trulsson L.M., Magnusson K.E., Timmerman H.M., Van Minnen L.P., Rijkers G.T., Gooszen H.G., Akkermans L.M. (2009). Probiotics prevent intestinal barrier dysfunction in acute pancreatitis in rats via induction of ileal mucosal glutathione biosynthesis. PLoS ONE.

[56] Caselli M., Cassol F., Calò G., Holton J., Zuliani G., Gasbarrini A. (2013). Actual concept of “probiotics”: Is it more fuctionals to science or business?. World J Gastroenterol..

[57] Dinan T.G., Cyran J.F. (2017). Gut instincts: Microbiota as a key regulator of brain development, ageing and neurodegeneration. J. Physiol..

[58] Schmidt C. (2015). Mental health: Thinking from the gut. Nature.

[59] Shaaban S.Y., El Gendy Y.G., Mehanna N.S., El Senousy W.M., El-Feki H.A.S., Saad K., El-Asheer O.M. (2018). The role of probiotics in children with autism spectrum disorder: A prospective, open-label study. Nutr. Neurosci..

[60] Sparrow S.S., Balla D.A., Cicchetti D.V. (1984). Vineland Adaptive Behavior Scales. American Guidance Service.

[61] Abidin R.R. (1995). Parenting Stress Index—Short Form.

[62] Schopler E., Lansing M.D., Reichler R.J., Marcus L.M. (2006). Pep3 Profilo Psicoeducativo.

[63] Goldstein S., Naglieri J.A., Giunti O.S. (2014). ASRS. Autism Spectrum Rating Scales.

[64] R Development Core Team (2015). R: A language and Environment for Statistical Computing.

[65] Harrell F.E.J. (2014). rms: Regression Modeling Strategies.

[66] McMurdie P.J., Holmes S. (2013). Phyloseq: An R package for reproducible interactive analysis and graphics of microbiome census data. PLoS ONE.

[67] Oksanen J. (2022). Vegan: Community Ecology Package; R Package Version 2.6-2. https://cran.r-project.org/web/packages/vegan/vegan.pdf.

[68] Rohart F., Gautier B., Singh A., Lê Cao K.-A. (2017). mixOmics: An R package for omics feature selection and multiple data integration. PLoS Comput. Biol..

[69] Lê Cao K.A., Costello M.-E., Lakis V.A., Bartolo F., Chua X.-Y., Brazeilles R., Rondeau P. (2016). MixMC: A multivariate statistical framework to gain insight into microbial communities. PLoS ONE.

[70] Higashida H., Furuhara K., Yamauchi A.M., Deguchi K., Harashima A., Munesue S., Lopatina O., Gerasimenko M., Salmina A.B., Zhang J.S. (2017). Intestinal transepithelial permeability of oxytocin into the blood is dependent on the receptor for advanced glycation end products in mice. Sci. Rep..

[71] Meyer-Lindenberg A., Domes G., Kirsch P., Heinrichs M. (2011). Oxytocin and vasopressin in the human brain: Social neuropeptides for translational medicine. Nat. Rev. Neurosci..

[72] Shahrestani S., Kemp A.H., Guastella A.J. (2013). The impact of a single administration of intranasal oxytocin on the recognition of basic emotions in humans: A meta-analysis. Neuropsychopharmacology.

[73] Sanctuary M.R., Kain J.N., Chen S.Y., Kalanetra K., Lemway D.G., Rose D.R., Yang H.T., Tancredi D.J., German J.B., Slupsky C.M. (2019). Pilot study of probiotc/colostrum supplementation on gut function in children with autism and gastrointestinal symptoms. PLoS ONE.

[74] Kaluzna-Czaplinska J., Blaszczyk S. (2012). The level of anabinitol in autistic children after probiotic therapy. Nutrition.

[75] West R., Roberts E., Sichel L.S., Sichel J. (2013). Improvements in gastrointestinal symptoms among children with autism spectrum disorder receiving the Delpro Probiotic and immunomodulator formulation. J. Prob. Health.

[76] Santocchi E., Guiducci L., Prosperi M., Calderoni S., Gaggini M., Apicella F., Tancredi R., Billeci L., Mastromarino P., Grossi E. (2020). Effects of probiotic supplementation on gatrointestinal, sensory and core symptoms in autism spectrum disorders: A randomized controlled trial. Front. Psychiatry.

[77] Siemann J.K., Muller C.L., Forsberg C.G., Blakely R.D., Veenstra-VanderWeele J., Wallace M.T. (2017). An autism-associated serotonin transporter variant disrupts multisensory processing. Transl. Psychiatri..

[78] Parracho H.M., Gibson G.R., Knott F., Bosscher D., Kleerebezem M., McCartney A.L. (2010). A double-blind, placebo-controlled, crossover-designed probiotic feeding study in children diagnosed with autistic spectrum disorders. Int. J. Probiotics Prebiotics.

[79] Slykerman R.F., Kang J., Van Zyl N., Barthow C., Wickens K., Stanley T., Coomarasamy C., Purdie G., Murphy R., Crane J. (2018). Effect of early probiotic supplementation on childhood cognition, behavior and mood a randomized, placebo-controlled trial. Acta Paediatr..

[80] Jones R.M., Carberry C., Hamo A., Lord C. (2017). Placebo-like response in absence of treatment in children with autism. Autism Res..

