# Supplementary material for: Randomized Double-Blind Crossover Study for Evaluating a Probiotic Mixture on Gastrointestinal and Behavioral Symptoms of Autistic Children
Source: J Clin Med. 2022 Sep 6;11(18):5263. doi: 10.3390/jcm11185263 (PMC9504504; doi:10.3390/jcm11185263)
Supplement: Supplementary file 1 [file jcm-11-05263-s001.zip › jcm-1838220-supplementary.pdf]

Supplementary Figures

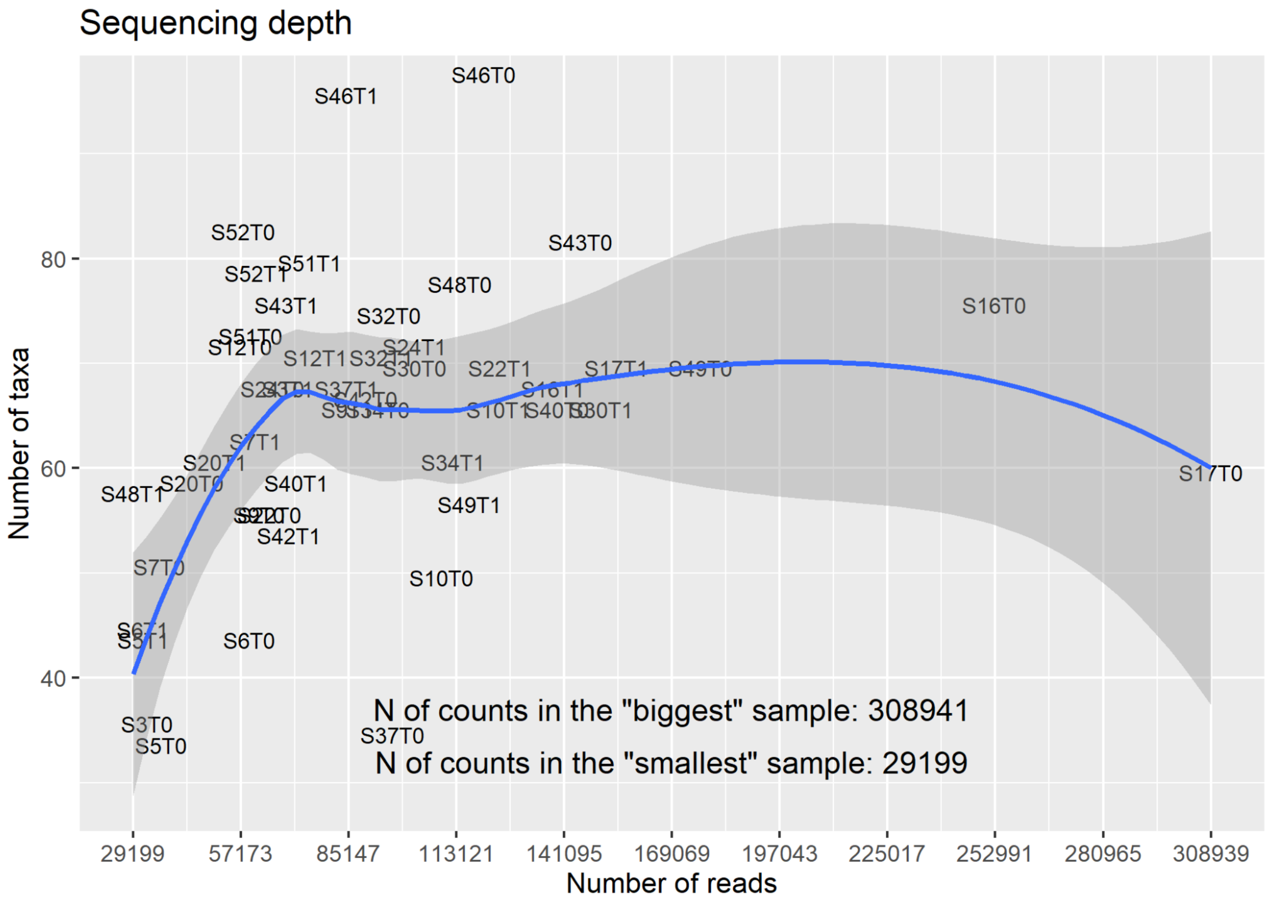

Figure S1: Number of taxa per number of reads representing the overall sequencing depth, before filtering.

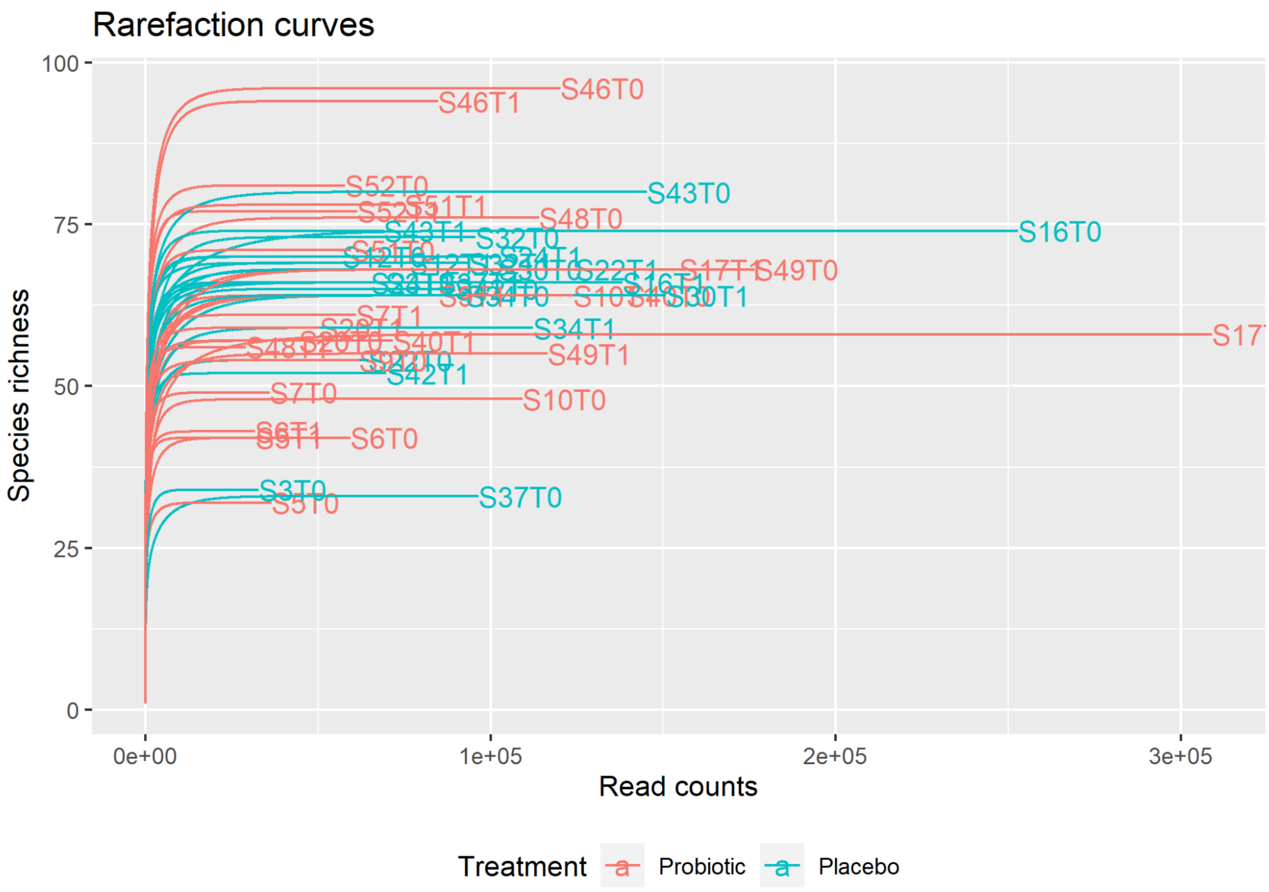

Figure S2: Rarefaction curves as a function of sampling depth.

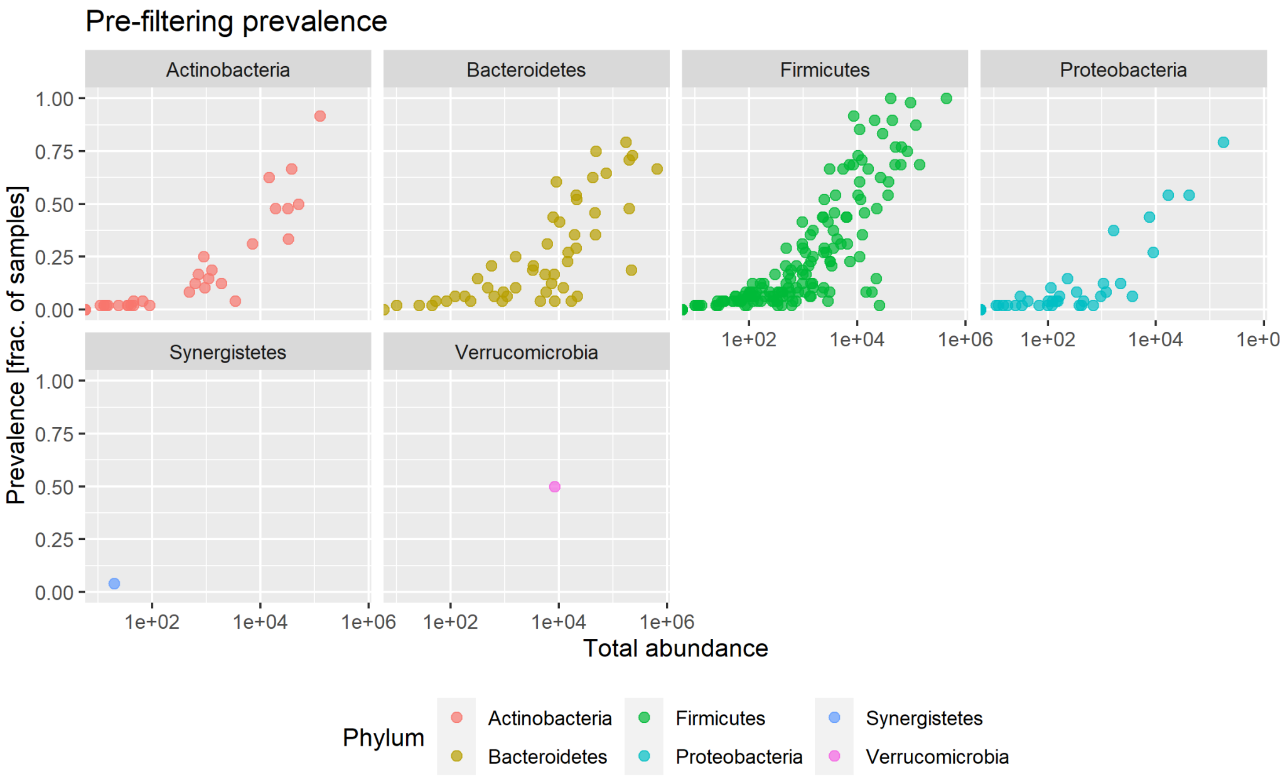

Figure S3: The 6 phyla detected in the dataset are depicted, plotted for their abundance scores and prevalence scores, accounting for the fraction of samples in which they were observed.

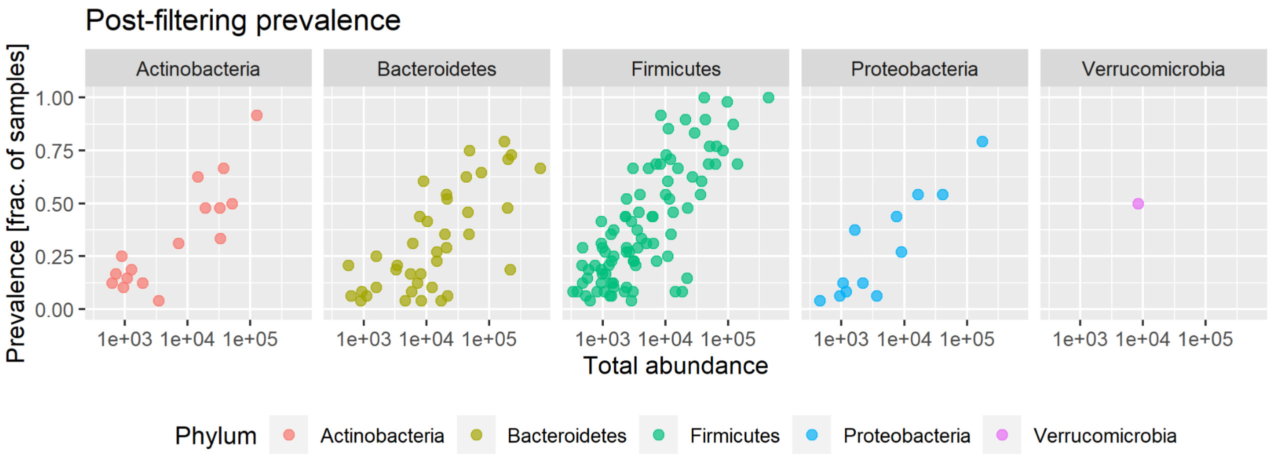

Figure S4: After a filter based on the relative abundance, data shows that 1 out of the initial 6 phyla were discarded, resulting in 5 phyla, which are present with a relative abundance of at least, 0,005%. These are plotted for their abundance scores and prevalence scores.
